# Alteronol Enhances the Anti-tumor Activity and Reduces the Toxicity of High-Dose Adriamycin in Breast Cancer

**DOI:** 10.3389/fphar.2019.00285

**Published:** 2019-03-29

**Authors:** Boxue Ren, Lei Ye, Jianwei Gong, Huanhuan Ren, Yangfang Ding, Xiaoyu Chen, Xiaona Liu, Peng Lu, Fei Wei, Wenjuan Xu, Qiusheng Zheng, Defang Li

**Affiliations:** ^1^School of Integrated Traditional Chinese and Western Medicine, Binzhou Medical University, Yantai, China; ^2^Key Laboratory of Xinjiang Phytomedicine Resource and Utilization, Ministry of Education, School of Pharmacy, Shihezi University, Shihezi, China; ^3^School of Rehabilitation Medicine, Binzhou Medical University, Yantai, China; ^4^Yantai Affiliated Hospital of Binzhou Medical University, Yantai, China; ^5^School of Public Health and Management, Binzhou Medical University, Yantai, China

**Keywords:** alteronol, adriamycin, breast cancer, cell cycle arrest, apoptosis, chemotherapy

## Abstract

The first-line chemotherapy drug adriamycin (ADM) is widely used for the treatment of breast cancer, but the acquired drug resistance and the normal tissue toxicity remain clinical challenges. Alteronol has been reported to exert wide-ranging anti-tumor activity. In this study, we firstly examined the synergistic anti-tumor effects and the underlying mechanisms of alteronol combined with ADM in breast cancer. We have found that the combination of alteronol and ADM significantly suppressed the expression levels of the cell cycle-related proteins (CDC2 and Cyclin B1) and induced cell cycle arrest at the G2/M phase, leading to cell proliferation inhibition in breast cancer 4T1 cells. Moreover, co-treatment of alteronol and ADM (i) remarkably activated p38 and JNK kinases, (ii) elevated ROS levels, (iii) triggered mitochondrial dysfunction, (iv) released cytochrome c into the cytoplasm, (v) upregulated apoptosis-related proteins, e.g., cleaved PARP, Bax, and cleaved caspase-3/9, and (vi) downregulated the expression of Bcl-2, followed by apoptosis. Furthermore, our *in vivo* studies showed that the low-dose combination of alteronol (2 mg/kg) and ADM (1 mg/kg) significantly inhibited tumor growth in tumor bearing mice, and the anti-tumor effect of the combination was the same as that of high-dose ADM (8 mg/kg). In addition, the low-dose combination group showed lower toxicities to major organs than the high-dose ADM group. Taken together, these data demonstrate that the low-dose combination of alteronol and ADM could notably improve the anti-tumor activity and have lower toxicities to major organs than those in high-dose ADM group.

## Introduction

Breast cancer is one of the most frequently occurring malignant tumors in women, originating in the epithelial tissue of the breast (Ferlay et al., 2015; Torre et al., 2015). It is characterized by strong invasion, a high recurrence rate, easy metastasis and poor prognosis (Weigelt et al., 2005). Clinical data have demonstrated that the incidence of breast cancer has been increasing in recent years. It has already threatened the public’s health and brought a tremendous economic burden to the society. Chemical therapy is an important treatment for breast cancer (Kuschel et al., 2000;Edlich et al., 2005), and adriamycin (ADM), a cytotoxic anthracycline, plays an important role in the treatment of breast cancer (Mccarthy et al., 2014). Up to now, ADM is recommended as the first-line chemotherapy for breast cancer treatment (Chen et al., 2017).

However, the acquired drug resistance to ADM in breast cancer treatment is a common phenomenon and a large obstacle, which limits its further application (Qiu et al., 2010). At the same time, it is reported that ADM can damage normal cells/tissues, e.g., the immune system and the heart. Therefore, effectively overcoming the problems of drug resistance and toxicity of ADM can improve the efficiency of breast cancer treatment. Drug combinations have become a main focus in the field of cancer therapy, as they can increase the sensitivity to chemotherapy and decrease the drug doses to overcome the adverse effects (Wei et al., 2015).

Alteronol is a novel compound isolated from the fermentation products of a microbial mutant strain in the bark of *Taxus chinensis (Pilger) Rehd* (Yao et al., 2012). Recently, several studies have shown that alteronol has anti-tumor effects in several types of neoplasms, such as leukemia (Liu Z.Z. et al., 2007), melanoma (Wang et al., 2014), gastric cancer (Liu X. et al., 2007), breast cancer (Ren et al., 2018), and prostate cancer (Yeung et al., 2012). These effects are related to the promotion of differentiation (Wang C. et al., 2015), the induction of apoptosis (Liu Z.Z. et al., 2007), cell cycle arrest (Liu X. et al., 2007), and the inhibition of invasion and metastasis (Wang et al., 2014). It has been reported that ADM also exerts anti-proliferative effects on breast cancer cells by inducing apoptosis and cell cycle arrest (Ku et al., 2015). However, the anti-tumor effect of the combination of alteronol and ADM on breast cancer has not been reported. In addition, understanding of the anti-tumor mechanisms of the combination of alteronol and ADM may provide novel insights for the clinical application in breast cancer.

In the current study, we firstly investigated whether alteronol combined with ADM synergistically enhances the anti-tumor effects, and the underlying mechanism, in breast cancer 4T1 cells. Then, we examined the anti-tumor growth effect and the normal cell/tissue toxicity of the combination in breast cancer bearing mice.

## Materials and Methods

### Cell Culture and Animals

Mouse breast cancer 4T1 cells were obtained from Cell Bank of the Committee on Type Culture Collection of the Chinese Academy of Sciences (Shanghai, China). The cell line was incubated in a humidified atmosphere of 37°C containing 5% CO_2_ with RPMI-1640 medium (Gibco Invitrogen, Grand Island, NE, United States) supplemented with 10% fetal bovine serum (TransGen Biotech Company Co., Ltd., Beijing, China) and 1% penicillin–streptomycin solution (HyClone, Los Angeles, CA, United States). BALB/c female mice (6–8 weeks old, 18–22 g) were purchased from the medical laboratory animal center of Xinjiang Medicine University (License No. SCXK (xin) 2017-0015). All the procedures involving animals were in accordance with the NIH guidelines for the care and use of laboratory animals and have been validated by the committees of animal ethics and experimental safety of Shihezi University.

### Measurement of Cell Viability

Cell viability assays were examined by the MTT assay. Cells were seeded into 96-well plates at a density of 1 × 10^5^ cells/mL and cultured at 37°C in a humidified incubator. After incubation for 24 h, cells were exposed to alteronol (≥99%, 20110711-01, ShantouStrand Biotech Co., Ltd., Shantou, China), ADM (H44024359, Shenzhen Main Luck Pharmaceuticals Inc., Shenzhen, China), or a combination of alteronol and ADM, at 37°C for 24 or 48 h. To each well 20 μL of 5 mg/mL MTT (Sigma-Aldrich, St. Louis, MO, United States) was added, and the cells were kept at 37°C for 4 h in the dark, as described previously (Gerlier and Thomasset, 1986; Hou et al., 2008). Then blue formazan crystals were dissolved with 150 μL dimethyl sulfoxide (DMSO) (Sigma-Aldrich, St. Louis, MO, United States). The optical density at 570 nm was measured using a microplate reader (Varioskan Flash 3001; Thermo Fisher Scientific, Inc., Waltham, MA, United States).

Following the Chou-Talalay combination index (CI) method (Chou, 2010), the CI was calculated for the evaluation of the effects of drug combinations, in order to examine the interaction between two drugs at different concentrations. The data were analyzed using CompuSyn software (Biosoft, Ferguson, MO, United States) to calculate the CI and DRI values. CI < 1, CI = 1, and CI > 1 indicate synergy, additivity, and antagonism, respectively.

### Determination of Morphological Alterations

The morphological changes of apoptotic cells were observed by Hoechst 33258 staining. The cells were placed in 6-well plates at 8 × 10^4^ cells/mL, exposed to the drug(s) for 48 h, and washed twice with ice-cold PBS. Cells were fixed with fixative consisting of methanol and glacial acetic acid (3:1, v/v) for 15 min, followed by Hoechst 33258 staining (10 mg/L) (Beijing Solarbio Science & Technology Co., Ltd., Beijing, China) for 10 min at room temperature and away from the light, as described previously (Soriano et al., 2013). Apoptosis morphology was captured using a fluorescence microscope (MIC00266; Zeiss, Germany) and analyzed by Image-Pro Plus Premier (version 9.1.4; Media Cybernetics Inc., Rockville, MD, United States).

### Cell Cycle Examination and Cell Apoptosis Assay by Flow Cytometry

The 4T1 cells (5 × 10^5^ cells/mL) were incubated with alteronol, ADM, or a combination at 37°C for 48 h, following which the cells were harvested and washed with PBS. Then the cells were prepared following the protocol of the Annexin V-FITC Apoptosis Detection Kit (Nanjing KeyGen Biotech Co., Ltd., Nanjing, China). In the cell cycle assay, the cells were dispersed, 85% methanol was added, and the cells were kept at −20°C overnight, resuspended in PBS containing propidium iodide (PI, 0.05 mg/mL) and RNase A (0.5 mg/mL), and subsequently incubated at 37°C for 30 min in the dark, as described previously (Hockenbery et al., 1990). Finally, the prepared cell samples were quantified using the FACScan flow cytometer (BD Biosciences, Franklin Lakes, NJ, United States) and analyzed by CellQuest^TM^ Pro acquisition software (BD FACSCalibur^TM^; San Jose, CA, United States).

### Analysis of Intracellular Reactive Oxygen Species (ROS) Levels

After the above indicated treatment for 48 h, the cells were collected and washed with ice-cold PBS. The cells were labeled with 30 μM DCFH-DA for 30 min at 37°C away from the light, following the protocol of the Reactive Oxygen Species Analysis Kit (Nanjing KeyGen Biotech Co., Ltd., Nanjing, China). Then the stained cells were analyzed under a flow cytometer (BD Biosciences, Franklin Lakes, NJ, United States). The fluorescence (excitation at 485 nm and emission at 525 nm) was monitored using a microplate reader (Varioskan Flash 3001; Thermo Fisher Scientific, Inc., Waltham, MA, United States).

### Measurement of Mitochondrial Membrane Potential (MMP)

Cells were exposed to alteronol, ADM, or a combination at 37°C for 48 h. Then the cells were collected and incubated with JC-1 (5,5′,6,6′-tetrachloro-1,1′,3,3′-tetraethyl benzimidazolyl carbocyanine iodide) for 20 min at 37°C in the dark, as described previously (Reers et al., 1991; Smiley et al., 1991). The incubated cells were resuspended in 1 × incubation buffer, followed by the analysis of JC-1 fluorescence using a FACScan Flow Cytometer (BD Biosciences, Franklin Lakes, NJ, United States). The fluorescence was measured at different excitation/emission wavelengths of 485/580 nm (red) and 485/530 nm (green) with a microplate reader (Varioskan Flash 3001; Thermo Fisher Scientific, Inc., Waltham, MA, United States).

### Western Blot Analysis

Total protein (50 μg) was extracted using cell lysis buffer (Beijing Solarbio Science & Technology Co., Ltd., Beijing, China), following the manufacturer’s instructions. The concentrations of total proteins were determined by the Bradford method. Then the proteins were separated by 8–15% sodium dodecyl sulphate-polyacrylamide gel electrophoresis (SDS-PAGE). After separation, the proteins were transferred to PVDF membranes (Millipore, Darmstadt, Germany), which were blocked for 1 h at room temperature. The PVDF membranes were incubated with primary antibodies (anti-Cyclin B1 [monoclonal, rabbit anti-mouse, 1:2000, Cell Signaling Technology, Beverly, MA, United States], anti-CDC2 [monoclonal, rabbit anti-mouse, 1:1000, Cell Signaling Technology, Beverly, MA, United States], anti-Bax [polyclonal, rabbit anti-mouse, 1:1000, Boster Biological Technology Co., Ltd., Wuhan, China], anti-Bcl-2 [monoclonal, rabbit anti-mouse, 1:400, Boster Biological Technology Co., Ltd., Wuhan, China], anti-PARP, anti-cleaved Caspase-3, anti-cleaved Caspase-9, and anti-JNK [polyclonal, rabbit anti-mouse, 1:500, Cell Signaling Technology, Beverly, MA, United States], anti-cytochrome c, anti-p-JNK, anti-p38, and anti-p-p38 [monoclonal, rabbit anti-mouse, 1:500, Cell Signaling Technology, Beverly, MA, United States], or anti-β-actin [monoclonal, mouse anti-mouse, 1:1000, ZSGB Bio-technology, Beijing, China]) at 4°C overnight. Next, the membranes were washed four times with TBST and incubated with HRP-conjugated goat anti-mouse/anti-rabbit secondary antibody (1:10,000, Beijing TransGen Biotech Co., Ltd., Beijing, China) for 1 h at room temperature. Finally, the protein bands were visualized using the SuperSignal^TM^ West Femto Trial Kit (Thermo Fisher Scientific, MA, United States) and subsequently imaged with a UVP chemiluminescence imaging system (Ultra-Violet Products Ltd., Cambridge, United Kingdom), followed by the analysis of the autoradiographs using ImageJ software (National Institutes of Health, United States). Results were normalized to the internal control β-actin. Three independent experiments were performed for each protein.

### Measurement of *in vivo* Anti-tumor Activity

The 4T1 cells (8 × 10^6^ cells/mL, 100 μL) were subcutaneously inoculated into the BALB/c mice. The formation of the tumors in BALB/c mice was monitored. When the tumors reached a size of 100 mm^3^, all inoculated mice were randomly divided into six groups (*n* = 6 for each group): (1) model group, (2) low-dose alteronol group, (3) low-dose ADM group, (4) combination group, (5) high-dose alteronol group, and (6) high-dose ADM group. The weight difference among groups was negligible. In the model group, an intraperitoneal injection of saline (10 ml/kg) was administered every 2 days. In groups 2 and 5, the mice were given 2 or 3 mg/kg of alteronol by intraperitoneal injection every other day, respectively. In groups 3 and 6, the mice were given of 1 or 8 mg/kg of ADM by intraperitoneal injection every week, respectively. In group 4 (the combination group), 2 mg/kg of alteronol and 1 mg/kg of ADM was administered every other day and every week, respectively. The weight of mice and volume of the tumor were recorded every day until the animals were sacrificed. The mice were sacrificed after the above treatments for 3 weeks, and the tumor and the serum of mice were collected.

### HE Staining

After the mice were sacrificed, the major organs (heart, liver, spleen, lung, and kidney) and tumor tissue were collected, washed with normal saline, and dried using tissue paper. Heart, liver, spleen, lung, kidney, and tumor tissue was fixed in 4% polyoxymethylene for 3 days and embedded in paraffin. The paraffin-embedded tissues were sectioned at 5 μm thickness and placed on slides. The paraffin sections were dried, deparaffinized, rehydrated, and washed. Then the sections were stained with hematoxylin and eosin (HE), dehydrated, and mounted with neutral resin. The HE-stained sections were observed under a microscope (Axio Imager. M2; Zeiss, Göttingen, Germany).

### Measurement of Cardiotoxicity, Hepatotoxicity, and Nephrotoxicity

After the mice were sacrificed, the serum of the mice was collected. The serum levels of creatine kinase (CK), lactate dehydrogenase (LDH), aspartate aminotransferase (AST), alanine aminotransferase (ALT), urea nitrogen (BUN), and creatinine (Cr) were measured using the respective assay kits, i.e., Creatine Kinase Assay Kit, Lactate Dehydrogenase Assay Kit, Aspartate Aminotransferase Assay Kit, Alanine Aminotransferase Assay Kit, Urea Assay Kit, and Creatinine Assay kit. All these assay kits were purchased from Nanjing Jiancheng Bioengineering Institute (Nanjing, China). CK and LDH were used for cardiac function evaluation, AST and ALT were employed for liver function examination, and BUN and Cr were used for renal function measurement.

### Statistical Analysis

Each experiment was carried out at least three times. All data are presented as mean ± standard deviation. The analyses were carried out using the SPSS 22.0 software package (version 22.0, SPSS Inc., SPSS, Chicago, IL, United States). One-way analysis of variance (ANOVA) and the Student *t*-test were used to calculate statistical significance. A value of *P* < 0.05 was chosen to indicate that the difference is statistically significant.

## Results

### The Synergism of Alteronol and ADM Against Breast Cancer 4T1 Cells

**FIGURE 1:**
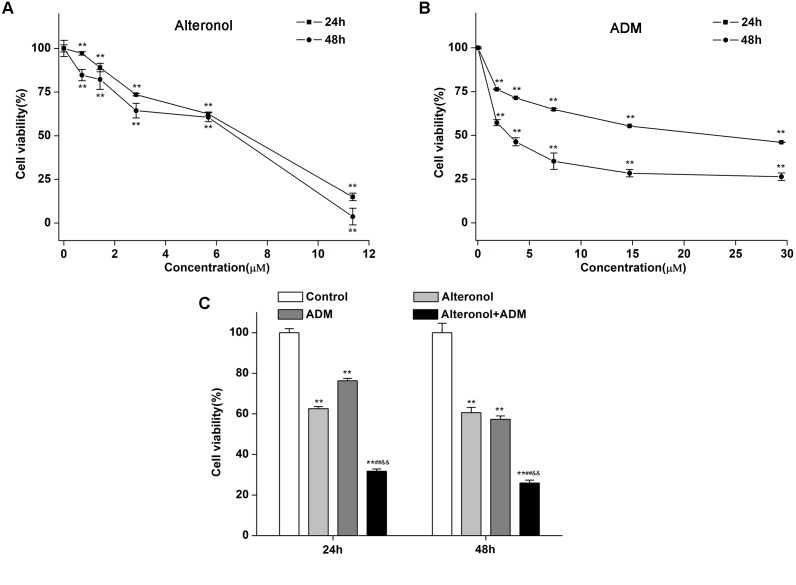
Combination of alteronol and ADM significantly inhibits cell viability in breast cancer 4T1 cells. **(A)** The anti-proliferative effect of alteronol on 4T1 cells was determined by MTT assay after 24 h or 48 h of treatment. **(B)** The anti-proliferative effect of ADM on 4T1 cells was determined by MTT assay after 24 h or 48 h of treatment. **(C)** After treatment with alteronol (5.68 μM), ADM (1.84 μM), or their combination for 24 or 48 h, respectively, the 4T1 cell viability was measured by MTT assay. ^∗^*P* < 0.05, ^∗∗^*P* < 0.01 vs. control group. ^#^*P* < 0.05, ^##^*P* < 0.01 vs. alteronol group; ^&^*P* < 0.05, ^&&^*P* < 0.01 vs. ADM group. All data are expressed as mean ± SD of three independent experiments.

To investigate whether alteronol could synergistically enhance the anti-proliferative activity of ADM *in vitro*, the cell survival of 4T1 cells was investigated by MTT assay after treatment with alteronol and/or ADM. 4T1 cells were firstly treated with different concentrations of alteronol (0∼11.36 μM) or ADM (0∼29.44 μM) for 24 and 48 h, respectively. We found that alteronol and ADM significantly inhibited the proliferation of 4T1 cells in a dose-dependent manner. IC_50_ values of alteronol were 6.87 and 5.62 μM after 24 and 48 h of treatment, respectively (Figure 1A), and IC_50_ values of ADM were 22.96 and 2.78 μM after 24 and 48 h of treatment, respectively (Figure 1B). Notably, the data indicate that alteronol and ADM both exert anti-proliferative effects on 4T1 cells and the effect after 48 h is the most significant.

We next investigated whether alteronol combined with ADM could have a synergistic effect. The MTT assay was used to measure the effect of alteronol combined with ADM on 4T1 cell proliferation. The Chou–Talalay CI method (Chou, 2010) and Compusyn software were used to calculate the CI and the dose reduction index (DRI). Our results show that alteronol synergistically enhanced the efficacy of ADM against breast cancer 4T1 cells (CI < 1) (Tables 1, 2). Furthermore, 4T1 cells were exposed to alteronol (5.68 μM), ADM (1.84 μM), and their combination at the same concentrations after 24 and 48 h. The combination of alteronol and ADM exerted stronger inhibitory effects than each single drug (Figure 1C).

**Table 1:** Synergistic analysis of the combination of alteronol and ADM on 4T1 cells.

Alteronol + adriamycin	Cell line	Parameter	Fa_30_	Fa_50_	Fa_70_	Fa_90_
	4T1	CI	4.58417	1.49738	0.67398	0.32756
		DRI (alteronol)	1.29891	1.67896	2.17022	3.26654
		DRI (adriamycin)	0.26217	1.10892	4.69044	46.6730

*Cells were treated with alteronol with or without ADM for 48 h. CI and DRI values were calculated by CompuSyn software. Fa, effect levels. Fa_30_–Fa_90_, 30–90% efficiency percentage. CI, combination index; CI = (D)_1_/(Dx)_1_ + (D)_2_/(Dx)_2_, where (Dx)_1_ and (Dx)_2_ represent the doses of drug 1 and 2 in a combination needed for achieving the same efficiency as that of the single drug 1 or 2 at D_1_ or D_2_, respectively, CI < 1,CI = 1, and CI > 1 indicate synergistic, additive, and antagonistic effects, respectively. Dose reduction index (DRI) indicates the fold change of the dose for each drug to be reduced in a combination compared to a single drug at a given effect. The greater the DRI value, the more the dose of a single drug should be reduced to achieve the same effect. The DRIs of alteronol and ADM are greater than 1, indicating a synergistic effect for the combined treatment.*

**Table 2:** CI of the combination of alteronol and ADM on 4T1 cells.

Concentration (μM)		4T1	
Alteronol	Adriamycin	CI	Fa
1.42+1.84		2.86421	0.35542
2.84+1.84		2.79825	0.39881
5.68+1.84		0.61315	0.74082
11.36+1.84		0.23669	0.94990

*CI, combination index; Fa, effect level.*

### The Combination of Alteronol and ADM Synergistically Induces Cell Cycle Arrest in 4T1 Cells

**FIGURE 2:**
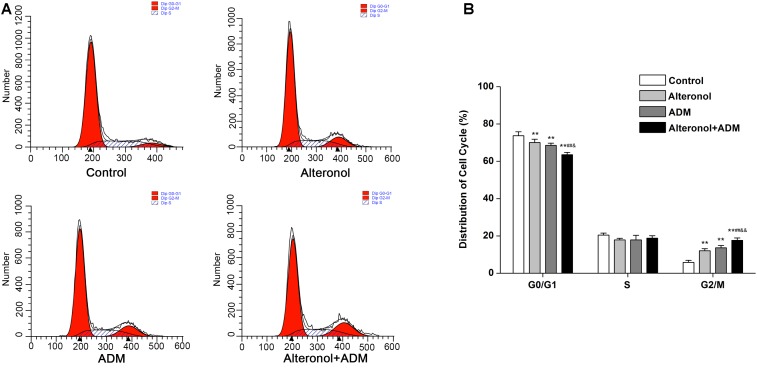
The effect of the combination of alteronol and ADM on cell cycle distribution in 4T1 cells. **(A)** Cell cycle distribution of 4T1 cells was determined by flow cytometry after treatment with alteronol, ADM, or both. **(B)** Quantitative analysis of the cell cycle distribution in 4T1 cells after the indicated treatments. ^∗^*P* < 0.05, ^∗∗^*P* < 0.01 vs. the control group. ^#^*P* < 0.05, ^##^*P* < 0.01 vs. alteronol group. ^&^*P* < 0.05, ^&&^*P* < 0.01 vs. ADM group. All data are expressed as mean ± SD of three independent experiments.

We further investigated the combined effect of alteronol (5.68 μM) and ADM (1.84 μM) on cell cycle progression. Our results show that, after alteronol and/or ADM treatment, the percentage of 4T1 cells in the G2/M phase was significantly higher in the treatment groups than in the control group (Figure 2A). Especially, co-treatment of alteronol and ADM caused a remarkable increase in the percentage of cells in the G2/M phase (17.63 ± 1.28%) compared with the single drug-treated groups (12.05 ± 1.10% for the alteronol group, 13.61 ± 1.27% for the ADM group) (Figure 2B). In contrast, the percentage of the cells in the G0/G1 phase was obviously decreased in the treatment groups when compared with the control group. After co-treatment, the percentage of cells in the G0/G1 phase was 63.56 ± 1.21%, whereas it was 70.06 ± 1.78% in the alteronol-treated group and 68.55 ± 1.21% in the ADM-treated group (Figure 2B). These data demonstrate that the combination of alteronol and ADM synergistically induces cell cycle arrest at the G2/M phase in 4T1 cells.

### The Combination of Alteronol and ADM Synergistically Regulates the Levels of G2 Phase Arrest-Related Proteins in 4T1 Cells

**FIGURE 3:**
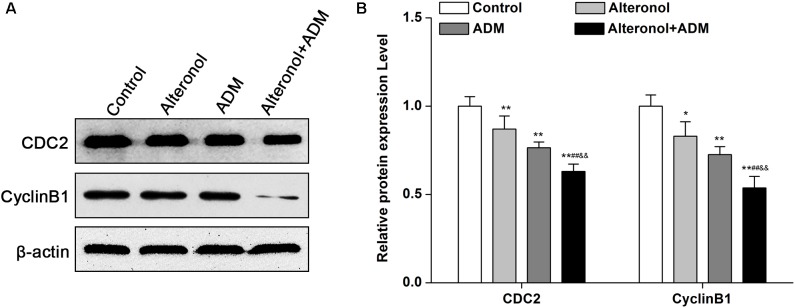
The co-treatment with alteronol and ADM regulates the levels of cell cycle-related molecules in 4T1 cells. **(A)** Representative images of the protein levels of CDC2 and Cyclin B1 detected by western blot. **(B)** Western blot analysis of CDC2 and Cyclin B1 protein levels after treatment with alteronol, ADM, or both, normalized to β-actin. ^∗^*P* < 0.05, ^∗∗^*P* < 0.01 vs. the control group. ^#^*P* < 0.05, ^##^*P* < 0.01 vs. alteronol group. ^&^*P* < 0.05, ^&&^*P* < 0.01 vs. ADM group. Data are presented as mean ± SD of three independent experiments.

After we observed that the combination of alteronol and ADM synergistically induced G2/M phase arrest, we detected the effect of alteronol, ADM, and their combination on the expression levels of the cell cycle-related proteins CDC2 and Cyclin B1. Western blot analysis showed that there was a remarkable decrease in CDC2 and Cyclin B1 proteins in drug treatment groups compared with the control group, especially in the co-treatment group (Figure 3A,B).

### The Combination of Alteronol and ADM Synergistically Induces Apoptosis in 4T1 Cells

**FIGURE 4:**
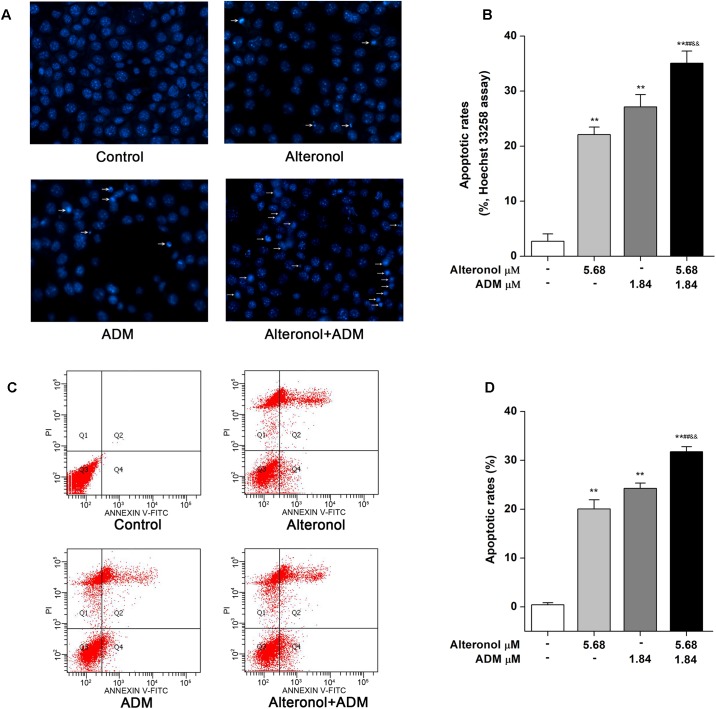
The effect of the combination of alteronol and ADM on apoptosis in 4T1 cells. **(A)** Morphological changes in 4T1 cells were examined by Hoechst 33258 staining after treatment with alteronol, ADM, or both. **(B)** Distribution of apoptosis rates in 4T1 cells as determined by Hoechst 33258 staining after the indicated treatments. **(C)** The apoptotic rates of 4T1 cells were detected by flow cytometry after the indicated treatments. **(D)** Quantitative analysis of apoptotic rates of 4T1 cells after the indicated treatments. ^∗^*P* < 0.05, ^∗∗^*P* < 0.01 vs. control group. ^#^*P* < 0.05, ^##^*P* < 0.01 vs. alteronol group. ^&^*P* < 0.05, ^&&^*P* < 0.01 vs. ADM group. All data are expressed as mean ± SD of three independent experiments.

As is well known, the induction of apoptosis has become an effective strategy for cancer treatment (Chu and Xing, 2008). To investigate whether the synergistic anti-proliferative effects of alteronol and ADM in 4T1 cells induce apoptosis, the morphological changes of 4T1 cells were examined by Hoechst 33258 staining. Condensed and bright chromatin, condensed nuclei, and nuclear fragmentation, as well as some cell debris, were observed in the drug-treated 4T1 cells (Figure 4A,B). Moreover, the above typical morphology of apoptosis remarkably increased in the combination group compared to the single drug-treated groups (Figure 4A,B). To confirm the above results, the apoptotic rates were further measured by flow cytometry after Annexin V-FITC/PI double staining. Flow cytometric analysis indicated that the apoptotic rate was significantly increased, i.e., from 0.41 ± 0.44% in the control group to 20.05 ± 1.88% in the alteronol-treated group, 24.25 ± 1.08% in the ADM-treated group, and 31.74 ± 1.03% in the combination group. The above data also show that the apoptotic rate of the combination group was significantly higher than those in the single drug-treated groups, implying that the combination of alteronol and ADM synergistically induces apoptosis in 4T1 cells (Figure 4C,D).

### The Combination of Alteronol and ADM Synergistically Regulates Apoptosis-Related Proteins in 4T1 Cells

**FIGURE 5:**
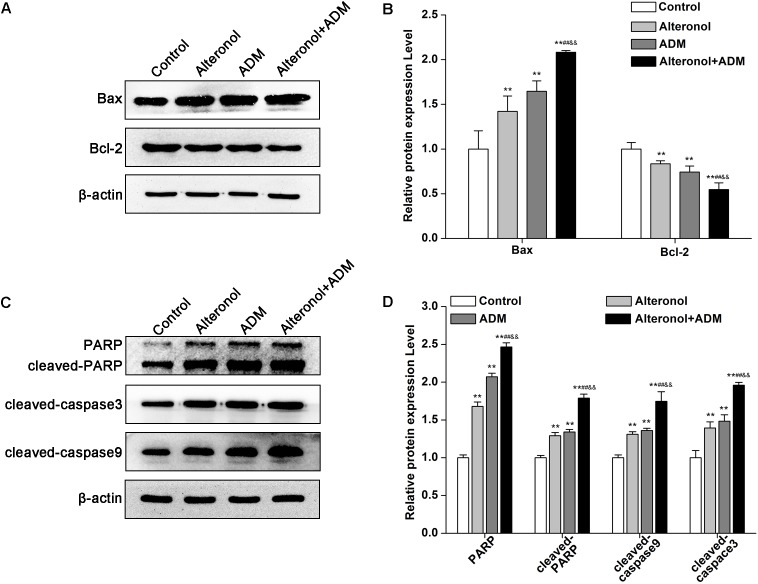
The effect of the combination of alteronol and ADM on protein levels of apoptosis-related molecules in 4T1 cells. **(A)** The protein levels of Bax and Bcl-2 were measured by western blot. **(B)** Quantitative analysis of Bax and Bcl-2 protein levels in 4T1 cells after treatment with alteronol and/or ADM. **(C)** The protein levels of cleaved PARP, cleaved caspase-9, and cleaved caspase-3 were examined by western blot. **(D)** Quantitative analysis of cleaved PARP, cleaved caspase-9, and cleaved caspase-3 protein levels after the indicated treatments. ^∗^*P* < 0.05, ^∗∗^*P* < 0.01 vs. control group. ^#^*P* < 0.05, ^##^*P* < 0.01 vs. alteronol group. ^&^*P* < 0.05, ^&&^*P* < 0.01 vs. ADM group. All data are expressed as mean ± SD of three independent experiments.

To further explore the mechanism underlying the anti-proliferative activity of alteronol and/or ADM in 4T1 cells, we analyzed apoptosis-related proteins after treatment with alteronol and/or ADM. We found that the protein level of Bax was notably upregulated, whereas the level of Bcl-2 was downregulated after treatment with alteronol and/or ADM (Figure 5A,B). Further study showed that the levels of cleaved PARP, cleaved Caspase-9, and cleaved Caspase-3 were upregulated following alteronol and/or ADM treatment (Figure 5C,D). Importantly, we found that the combination of alteronol and ADM had the strongest regulatory effect on the levels of apoptosis-related proteins (Figure 5A–D). Collectively, these data further confirm the synergistic and anti-proliferative effect of the combination of alteronol and ADM during 4T1 cell apoptosis.

### The Combination of Alteronol and ADM Synergistically Increases the ROS Levels and Activates the Mitochondrial Apoptotic Pathway in 4T1 Cells

**FIGURE 6:**
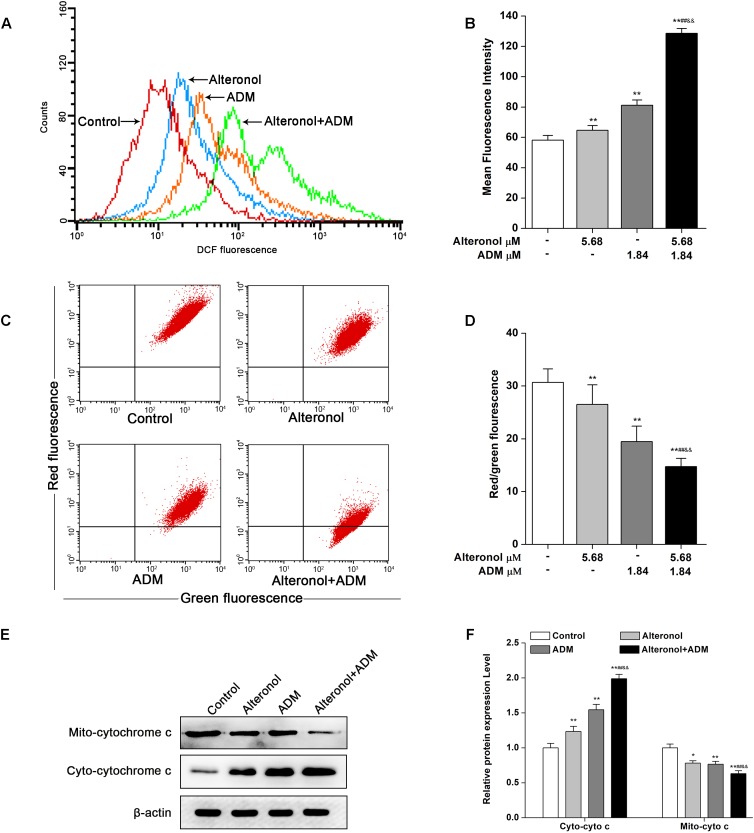
Co-treatment with alteronol and ADM triggers ROS generation and MMP loss in 4T1 cells. **(A)** The ROS levels in 4T1 cells were evaluated by flow cytometry after treatment with alteronol and/or ADM. The ROS levels are indicated by DCF fluorescence. **(B)** Quantitative analysis of the fluorescence intensity of DCF after treatment with alteronol and/or ADM. **(C)** The representative images of MMP in 4T1 cells using JC-1 staining after the indicated treatments. **(D)** Quantitative analysis of the ratio of red fluorescence to green fluorescence after alteronol and/or ADM treatment. **(E)** The protein levels of cytoplasmic cytochrome c as analyzed by western blot. **(F)** Quantitative analysis of the protein levels of cytoplasmic cytochrome c. The relative protein levels of cytoplasmic cytochrome c were normalized to the value in the control group. ^∗^*P* < 0.05, ^∗∗^*P* < 0.01 vs. the control group. ^#^*P* < 0.05, ^##^*P* < 0.01 vs. alteronol group. ^&^*P* < 0.05, ^&&^*P* < 0.01 vs. ADM group. All data are presented as mean ± SD of three independent experiments.

To examine whether alteronol and/or ADM could trigger ROS generation and activation of the mitochondrial apoptotic pathway in 4T1 cells, we examined the ROS levels by DCFH-DA staining and the loss of mitochondrial membrane potential (MMP, ΔΨm) by JC-1 dye. We found that the intracellular ROS levels were significantly elevated in 4T1 cells after treatment with alteronol and/or ADM when compared to the control group, especially in the combination group (Figure 6A,B). Moreover, in comparison with the control group, treatment with alteronol and/or ADM caused a notable decrease in the ratio of red and green fluorescence, indicating that alteronol and/or ADM could reduce the MMP in 4T1 cells (Figure 6C,D). Meanwhile, the combination of alteronol and ADM obviously reduced the MMP when compared to the single drug-treated groups (Figure 6C,D). Considering that the loss of MMP may affect the permeability of the mitochondrial membrane, which can lead to leakage of cytochrome c into the cytoplasm (Kinnally and Antonsson, 2007), the level of cytoplasmic cytochrome c (cyto-cytochrome c) was examined by western blot. We found that the cyto-cytochrome c level was dramatically increased in 4T1 cells after treatment with alteronol and/or ADM, and the cyto-cytochrome c level in the combination group was significantly higher than that in the single drug-treated groups (Figure 6E,F). These results imply that the combination of alteronol and ADM synergistically increased the ROS levels and induced the activation of the mitochondrial apoptotic pathway in 4T1 cells.

### The Combination of Alteronol and ADM Synergistically Induces p38 and JNK Activation in 4T1 Cells

**FIGURE 7:**
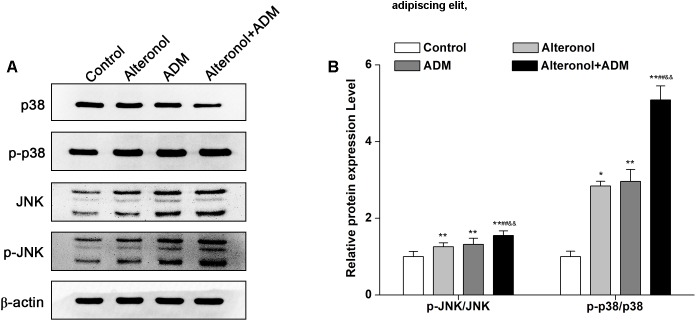
The effect of the combination of alteronol and ADM on the protein levels of MAPK-related molecules in 4T1 cells. **(A)** The protein levels of MAPK-associated molecules as detected by western blot. **(B)** Western blot analysis of the protein levels of MAPK-associated molecules normalized by β-actin. ^∗^*P* < 0.05, ^∗∗^*P* < 0.01 vs. the control group. ^#^*P* < 0.05, ^##^*P* < 0.01 vs. alteronol group. ^&^*P* < 0.05, ^&&^*P* < 0.01 vs. ADM group. Data are presented as mean ± SD of three independent experiments.

To explore whether the combination of alteronol and ADM inhibits cell proliferation via the mitogen-activated protein kinase (MAPK) signaling pathway, the expression levels of MAPK signaling molecules, such as p38 MAPK kinase and c-Jun amino-terminal kinase (JNK), were measured by western blot, and the signals were quantified by densitometry. Western blot analysis demonstrated that the protein levels of p-p38, JNK, and p-JNK were significantly elevated in treatment groups compared to the control group. Moreover, they were significantly higher in the combination group compared to the single drug-treated groups (Figure 7A,B). However, alteronol and/or ADM remarkably decreased the p38 protein level in 4T1 cells, and the p38 level in the combination group was more significantly decreased than in the single-drug administrated group (Figure 7A,B). These data suggest that alteronol and ADM could synergistically induce MAPK/p38 and MAPK/JNK activation.

### The Combination of Alteronol and ADM Synergistically Inhibits Tumor Growth *in vivo* and Simultaneously Reduces the Toxicity Induced by High-Dose ADM

**FIGURE 8:**
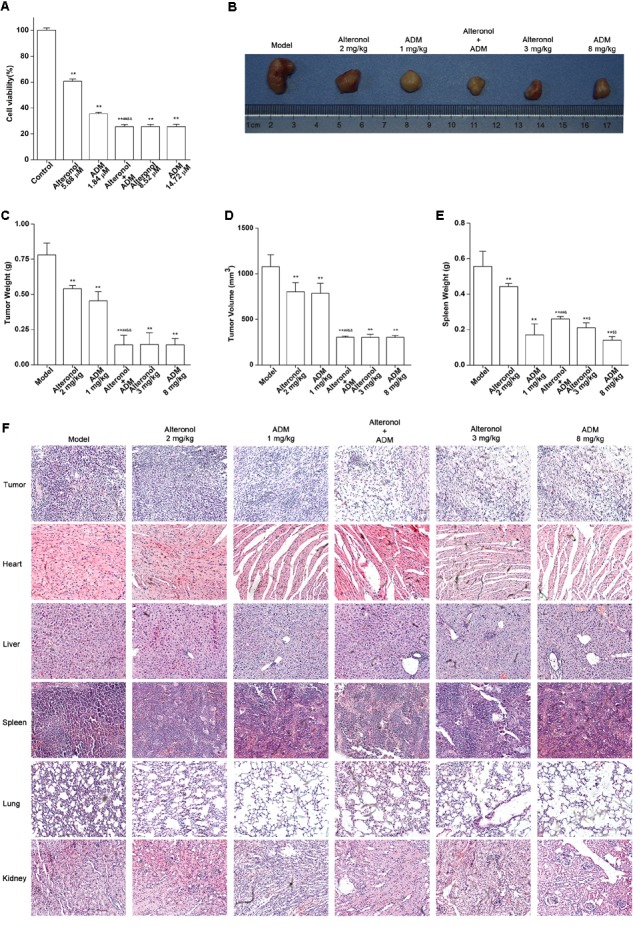
The anti-tumor effect of the combination of alteronol and ADM *in vivo*. **(A)** The cell viability was measured by MTT assay after treatment with different doses of alteronol (5.68 and 8.52 μM) and/or ADM (1.84 and 14.72 μM) for 48 h. **(B)** Microscopic view of tumor tissue in mice. **(C)** The tumor weights isolated from breast cancer 4T1 cell bearing mice after the indicated treatments for 2 weeks. **(D)** Isolated tumor volumes from the tumor bearing mice. **(E)** Isolated spleen weight from breast cancer 4T1 cell bearing model. **(F)** Histological assessments of tissues using HE staining after treatment with alteronol, ADM, or both. ^∗^*P* < 0.05, ^∗∗^*P* < 0.01 vs. model group. ^#^*P* < 0.05, ^##^*P* < 0.01 vs. alteronol group (2 mg/kg). ^&^*P* < 0.05, ^&&^*P* < 0.01 vs. ADM group (1 mg/kg). ^$^*P* < 0.05, ^$$^*P* < 0.01 vs. the low-dose combination group.

Taking into account the results obtained *in vitro* experiments, we subsequently evaluated the anti-tumor effect and the toxicity of the combination of alteronol and ADM in a tumor bearing mouse model. Firstly, MTT assay data showed that the anti-proliferation effect of high-dose alteronol (8.52 μM) or high-dose ADM (14.72 μM) was lower than the anti-proliferation effect of the low-dose combination of alteronol (5.68 μM) and ADM (1.84 μM) (Figure 8A). Then, based on the drug concentrations in the MTT assay and our previous preliminary experiments, we examined the anti-tumor effects of intraperitoneal injections of alteronol (2 and 3 mg/kg) and/or ADM (1 and 8 mg/kg) in breast cancer 4T1 cells bearing BALB/c mice. We found that all treatments markedly inhibited tumor growth (e.g., tumor weight and tumor volume) when compared to the model group (Figure 8B–D). Interestingly, the anti-tumor effect of the low-dose combination treatment was more effective than the single low-dose alteronol/ADM treatment groups, and the low-dose combination treatment had the same anti-tumor effect as the high-dose alteronol/ADM treatment groups, implying that alteronol enhanced the anti-tumor effect of ADM in breast cancer (Figure 8B–D).

**FIGURE 9:**
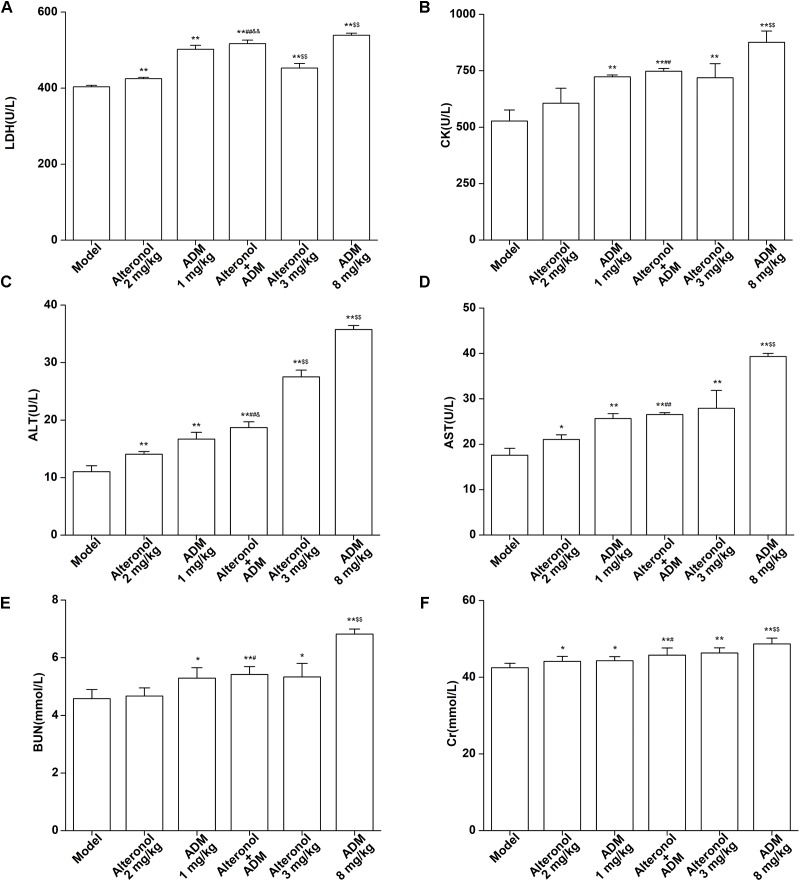
Effect of the combination of alteronol and ADM on the heart/liver/kidney function markers *in vivo*. Effects of alteronol (2 mg/kg and 3 mg/kg), ADM (1 mg/kg and 8 mg/kg), or a combination on the levels of **(A)** LDH, **(B)** CK, **(C)** ALT, **(D)** AST, **(E)** BUN, and **(F)** Cr in mouse serum. ^∗^*P* < 0.05, ^∗∗^*P* < 0.01, vs. model group. ^#^*P* < 0.05, ^##^*P* < 0.01, vs. alteronol group (2 mg/kg); ^&^*P* < 0.05, ^&&^*P* < 0.01, vs. ADM group (1 mg/kg); ^$^*P* < 0.05, ^$$^*P* < 0.01, vs. the low-dose combination group.

The spleen is an important immune organ of the body and is highly sensitive to cytotoxic drugs, because chemotherapy drugs induce spleen atrophy and inhibit the immune function of the body (Hadden, 2003; Kastelan et al., 2003). Therefore, the spleen index can reflect the immune function of the body. We found that the weight of the spleen isolated from the low-dose combination-treated breast cancer bearing mice was higher than that in the high-dose (8 mg/kg) ADM-treated group, indicating that the low-dose combination of alteronol with ADM is less toxic than a high dose of ADM (Figure 8E). Furthermore, the ADM-related toxicity to major organs was examined by HE staining and serum biochemical assays. We found some serious damage to the heart, liver, spleen, lungs, and kidney in the high-dose ADM-treated (8 mg/kg) group. However, co-treatment had very lower toxicities to these tissues compared to the high-dose ADM-treated (8 mg/kg) group (Figure 8F). Consistently, the serum levels of LDH, CK, ALT, AST, BUN, and Cr in the high-dose ADM-treated (8 mg/kg) group were significantly higher than those in the low-dose combination-treated group, indicating that the low-dose combination of alteronol with ADM had lower toxicities to the heart, kidney, and liver when compared to those in the high-dose ADM (8 mg/kg) group (Figure 9A–F).

## Discussion

Clinical data showed that ADM has a significant effect on many kinds of tumors, and it is widely used in the clinical chemotherapy of breast cancer (Mccarthy et al., 2014). However, it is toxic and susceptible to drug resistance at high doses, which is one of the important reasons for the failure of breast cancer chemotherapy (Pie et al., 2007; Wang X. et al., 2015). Therefore, the reduction of the concentration and toxicity of ADM under the premise of ensuring the therapeutic effect is a key problem in the treatment of breast cancer. Recently, many kinds of combined drugs are commonly used in the treatment of tumors, and good curative effects have been achieved (Zheng et al., 2018). Treatment with a combination of ADM and Taxol (paclitaxel) is a current clinical recommendation (Ardavanis et al., 2008), but its application is hampered by the collection of the raw material for paclitaxel, whereby rare plant resources are destroyed (Gelderblom et al., 2001). Therefore, the most important thing is to find a substitute for paclitaxel.

Alteronol is a novel compound that is attracting much attention globally and exhibits promising anti-tumor properties (Yao et al., 2012). Previous studies have demonstrated that alteronol exerts anti-tumor effects in several types of cancer, such as prostate cancer (Yeung et al., 2012), gastric cancer (Liu X. et al., 2007), breast cancer (Wang et al., 2013), and melanoma (Wang et al., 2014; Wang C. et al., 2015). However, there have been no reports on the comprehensive analysis of the effect of the combination of alteronol and ADM on breast cancer using both *in vitro* and *in vivo* models. In this study, we found that alteronol can synergistically enhance the anti-tumor effect of ADM against breast cancer through inducing cell cycle arrest at the G2/M phase and apoptosis.

Cell cycle regulation is one of the main mechanisms behind the anti-proliferation effects of many drugs in cancer cells (Ke et al., 2016). Cyclin B1 is an important inhibitory factor at the G2/M phase; the formation of a Cyclin B1/CDC2 complex promotes cell cycle progression (Pyo et al., 2013). Our data show that the combination of alteronol and ADM synergistically downregulated the expression of Cyclin B1 and CDC2, consequently inducing cell cycle arrest at the G2/M phase.

Many studies have confirmed that when ROS levels reach a certain threshold, this can lead to mitochondrial dysfunction and a reduction of MMP, resulting in the release of cytochrome c into the cytoplasm, the activation of Bax/Bcl-2, caspase-3, and caspase-9, and subsequently the cleavage of PARP, thereby inducing an endogenous apoptosis pathway (Wu et al., 2010; Russo et al., 2012; Cooley-Andrade et al., 2016). Indeed, we found that the combination of alteronol and ADM synergistically elevated ROS levels, triggered mitochondrial dysfunction, released cytochrome c into the cytoplasm, and elevated the levels of apoptosis-related proteins, e.g., the Bax/Bcl-2 ratio, cleaved PARP, and cleaved caspase-3/9, thus eventually inducing breast cancer 4T1 cell apoptosis.

It has been reported that JNK and p38 MAPK belong to the MAPK family (Ji et al., 2009), and a growing body of evidence suggests that activation of p38 MAPK and JNK plays a critical role in the cell cycle and cell proliferation, differentiation, survival, and death (Aw, 2010; Ray et al., 2012). The MAPK pathway can be activated by many factors and is involved in extrinsic apoptosis pathways, such as the death receptor pathway and the mitochondrial pathway (Weston and Davis, 2002). Some studies demonstrated that many chemotherapeutic drugs can activate JNK, a pro-apoptotic kinase, to promote apoptosis (Lei and Davis, 2003). JNK can further activate cytochrome c, after JNK enters the mitochondrion, which is the initiation of the mitochondrial pathway (Zhao et al., 2014). It has been proved that the activation of p38 can promote cell death (Yosimichi et al., 2001). Consistent with these reports, the combination of alteronol and ADM synergistically induced the activation of p38 and JNK and subsequently promoted breast cancer 4T1 cell apoptosis.

Finally, a tumor bearing mouse model was employed to evaluate the *in vivo* anti-tumor effect of the combination of alteronol and ADM. We found that the low-dose combination of alteronol (2 mg/kg) and ADM (1 mg/kg) has the same anti-tumor effect as high-dose ADM (8 mg/kg) and synergistically inhibited tumor growth in the tumor bearing mice. Meanwhile, when compared to the high-dose ADM group, the low-dose combination group showed less toxicity to major organs due to the low dose of ADM (1 mg/kg).

However, there are some limitations in this study. First, the evaluation of the anti-tumor effect of the combination of alteronol and ADM was restricted to one mouse breast cancer cell line, 4T1, and thus other human breast cancer (e.g., MCF-7 or/and MDA-MB-231) cells could also be employed to examine the anti-tumor effect. Second, the toxicity of alteronol and/or ADM to major organs was evaluated in breast cancer bearing mice, so our research could be expanded to the *in vitro* cytotoxicity of alteronol and/or ADM in cardiomyocytes and hepatocytes.

## Conclusion

In summary, in the present study we found that the combination of alteronol and ADM could synergistically enhance the anti-tumor affect and reduce the toxicity of high-dose ADM, indicating that alteronol and ADM represent a novel anti-tumor drug combination candidate against breast cancer.

## Data Availability

All datasets generated for this study are included in the manuscript and/or the supplementary files.

## Ethics Statement

This study was carried out in accordance with the recommendations of the First Affiliated Hospital of Shihezi University of guidelines, the First Affiliated Hospital of Shihezi University of committee. The protocol was approved by the First Affiliated Hospital of Shihezi University of committee.

## Author Contributions

BR, LY, QZ, and DL designed the study. BR and LY wrote the manuscript. BR, LY, JG, HR, YD, XC, XL, PL, FW, and WX conducted experiments and analyzed data. BR, LY, QZ, and DL contributed to manuscript drafting. All authors read and approved the final manuscript.

## Conflict of Interest Statement

The authors declare that the research was conducted in the absence of any commercial or financial relationships that could be construed as a potential conflict of interest.
